# The Impact of Fly Ash on the Properties of Cementitious Materials Based on Slag-Steel Slag-Gypsum Solid Waste

**DOI:** 10.3390/ma17194696

**Published:** 2024-09-24

**Authors:** Fei Wang, Huihui Du, Zhong Zheng, Dong Xu, Ying Wang, Ning Li, Wen Ni, Chao Ren

**Affiliations:** 1 College of Materials Science and Engineering, Chongqing University, Chongqing 400044, China; 2 Shougang Jingtang Iron&Steel Co., Ltd., Tangshan 063200, China; 3 Shougang Group Research Institute of Technology, Beijing 100043, China; 4 Nanjing Institute of Environmental Sciences, Ministry of Ecology and Environment, Nanjing 210042, China; 5 College of Engineering, University of Miami, Miami, FL 33146, USA; 6 School of Civil and Engineering, University of Science and Technology Beijing, Beijing 100083, China; 7 School of Civil and Engineering, Tangshan University, Tangshan 063002, China

**Keywords:** fly ash, steel slag, solid waste-based cementitious materials, ettringite (AFt), C-(A)-S-H gel

## Abstract

This paper presents a novel low-carbon binder formulated from fly ash (FA), ground granulated blast furnace slag, steel slag, and desulfurization gypsum as a quaternary solid waste-based material. It specifically examines the influence of FA content on the mechanical properties and hydration reactions of the quaternary solid waste-based binder. The mortar test results indicate that the optimal FA content is 10%, which yields a 28-day compressive strength 11.28% higher than that of the control group without FA. The spherical particles of fly ash reduce the overall water demand and provide a “lubricating” effect to the paste due to their continuous gradation, improving the fluidity of the slag-steel slag-gypsum cementitious materials. The micro test results indicate that fly ash has minimal effect on the early hydration products and process of the solid waste-based cementitious materials, but after 7 days, it continuously dissolves silicon-oxygen tetrahedrons or aluminum-oxygen tetrahedrons, consuming Ca^2+^ and OH^−^ in the system. After 28 days, the amount of ettringite and C-(A)-S-H gel generated increases significantly. The pozzolanic activity of fly ash is mainly stimulated by the Ca(OH)_2_ from steel slag in the later hydration stage. Additionally, spherical fly ash particles can fill the voids in the hardened paste, reducing the formation of cracks and weak zones, and thereby contributing to a denser overall structure of the hydrated binder. The findings of this paper provide data support for the development of low-carbon cement-free binders using fly ash in conjunction with metallurgical slags, thereby contributing to the low-carbon advancement of the construction materials industry.

## 1. Introduction

As the global population continues to grow and urbanization progresses, there remains a sustained and substantial demand for construction materials, notably concrete, a trend expected to persist for several decades [1]. However, the production and use of materials such as concrete consume vast amounts of natural resources and release significant quantities of carbon dioxide. Cement, in particular, which serves as the binder in concrete, is one of the leading contributors to anthropogenic carbon emissions worldwide [2]. In response to the global challenge of mitigating greenhouse gas emissions and the resulting rise in global temperatures, the search for low-carbon alternatives to cement has emerged as a prominent area of research in the field of construction materials [3]. A key strategy in this endeavor is the development of low-carbon concrete by substituting cement with solid waste materials [4]. Among the more mature technologies currently under investigation and application is the partial replacement of cement with supplementary cementitious materials such as ground granulated blast furnace slag (GGBS), fly ash (FA), and silica fume in concrete production [5].

However, as the global imperative to curb greenhouse gas emissions intensifies, the development of novel low-carbon construction materials has become increasingly urgent. These new materials are characterized by several key features: first, their low carbon footprint [6]; second, the exclusive use of solid waste as raw materials [7]; and third, their mechanical properties are comparable to or significantly surpass those of traditional cement products. For instance, researchers have explored the use of waste glass and waste rubber in concrete production, resulting in enhanced macroscopic performance of the corresponding construction materials while also achieving lower carbon emissions [8,9]. Of particular promise is the collaborative utilization of various industrial solid wastes, such as GGBS, steel slag (SS), desulfurization gypsum (DG), and red mud, to develop cement-free, low-carbon binders. Studies indicate that these materials exhibit mechanical properties at least equivalent to those of cement, with carbon emissions reduced to merely 10% of those associated with conventional cement production [10,11,12,13].

Among the various solid wastes, fly ash is regarded as one of the most promising materials for replacing cement due to its favorable particle size distribution and high pozzolanic activity [14]. Fly ash is an industrial waste residue discharged from coal-fired power plants, known as an “urban mineral”. In 2020 alone, the annual discharge of fly ash in China reached 650 million tons. Fly ash contains a large amount of silicate and aluminate glass, which have potential pozzolanic activity [15]. In an alkaline environment, fly ash can undergo hydration reactions to produce cementitious calcium silicate hydrate and calcium aluminate hydrate, making it suitable for use in building materials. Currently, fly ash can be used in alkali-activated cementitious materials, fillers, and concrete admixtures [16,17,18]. When used in fillers, fly ash can reduce the amount of cement needed and enhance the solidification ability of the binder [19,20]. Additionally, as spherical particles, fly ash exhibits filling, micro-aggregate, and activity effects when mixed into cementitious materials or concrete, improving the mechanical properties and microstructure of the cementitious materials [21,22]. Furthermore, researchers have identified that the use of fly ash in the production of high-strength concrete (HSC) offers additional advantages, including the improvement of porosity and the reduction of heat of hydration [23].

Although extensive research and application have focused on the use of fly ash in cement-based materials, there is a relative scarcity of studies on the synergistic utilization of fly ash with other solid wastes for the development of novel low-carbon materials. In the context of co-processing multiple solid wastes to create these new low-carbon materials, steel slag is of particular interest due to its high calcium hydroxide content. The high alkalinity of calcium hydroxide in steel slag can effectively activate the pozzolanic reactivity of fly ash, generating C-S-H gel and providing certain mechanical properties [24,25]. Steel slag-slag-gypsum solid waste-based cementitious materials have the problem of low early strength [26]. Additionally, research by Li Ying et al. [10] found that the Ca/Si ratio of the calcium silicate hydrate gel generated during the hydration of steel slag-slag-gypsum solid waste-based cementitious materials is low, and highly polymerized C-S-H gel has strong mechanical properties. However, a notable challenge with SS-GGBS-DG-based solid waste binders is their low early strength [27]. Regarding fly ash, the performance characteristics and hydration mechanisms when used synergistically with materials such as GGBS and SS to develop novel cement-free binders remain unclear.

This study investigates the impact of fly ash on the macro- and micro-properties of SS-GGBS-DG-based ternary solid waste binders. Building upon the existing ternary solid-waste binders system, the research employs XRD, TG, ICP, and SEM techniques to analyze the hydration mechanism of a novel quaternary binder composed of FA, SS GGBS, and DG. The findings of this study are expected to provide valuable data supporting the application of fly ash in new low-carbon binders, while also addressing performance deficiencies in the SS-GGBS-DG system. By advancing the synergistic utilization of multiple solid wastes and contributing to carbon emission reduction in the construction industry, this research holds significant economic and environmental benefits.

## 2. Materials

**Figure 1:**
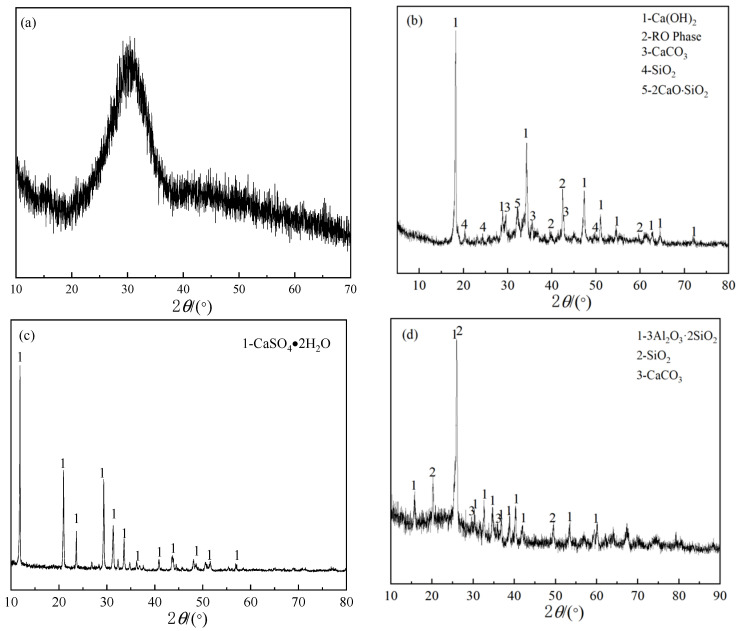
XRD patterns of the raw materials. (**a**) slag, (**b**) steel slag, (**c**) desulfurization gypsum, (**d**) fly ash.

The cementitious materials used in the experiment were prepared from slag, steel slag, desulfurization gypsum, and fly ash, all provided by Shougang Jingtang Iron and Steel United Co., Ltd. (Tangshan, China) The sand used was ISO standard sand, purchased from China Building Materials Research Institute. The standard sand used in this study conforms to the requirements for sand used in mortar specimens as specified in ISO 679:2009 [28]. The admixture used was a polycarboxylate superplasticizer, purchased from Beijing Muhu Admixture Co., Ltd. (Beijing, China) The chemical composition of the raw materials for the cementitious materials is shown in Table 1, and the X-ray diffraction (XRD) patterns are shown in Figure 1.

The quality coefficient K of the slag is 1.84, meeting the quality requirements for Grade A slag as specified in the GB/T 203-2008 standard [29] for granulated blast furnace slag used in cement. The XRD pattern of the slag, shown in Figure 1a exhibits a broad peak between 23° and 34°, indicating that the slag is primarily in an amorphous glassy state. The steel slag, which is desulfurization slag produced during the pre-desulfurization stage of molten iron, has an XRD pattern shown in Figure 1b [30]. Its main mineral phases are calcium hydroxide (Ca(OH)_2_), dicalcium silicate (Ca_2_SiO_4_), calcium ferrite phases (Ca_2_Fe_2_O_5_, CaFeO_2_), and the RO phase. The XRD pattern of desulfurization gypsum, shown in Figure 1c, indicates that its main mineral phase is gypsum (CaSO_4_·2H_2_O). The XRD pattern of fly ash, shown in Figure 1d, reveals that its main mineral phases are mullite (3Al_2_O_3_·2SiO_2_), quartz (SiO_2_), and calcite (CaCO_3_).

**Figure 2:**
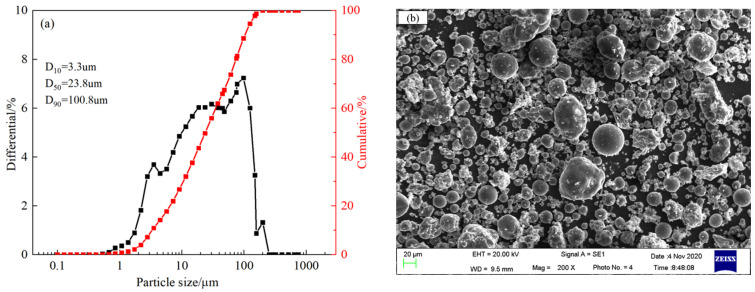
Particle size distribution and microscopic morphology of fly ash raw material. (**a**) Particle size distribution, (**b**) SEM morphology.

Figure 2 shows the particle size distribution and microstructure of the fly ash raw material. The fly ash exhibits a well-distributed particle size, with a specific surface area of approximately 342 m^2^/kg. The residue on a 45 μm square hole sieve is 24%, meeting the standards for Grade II fly ash. Additionally, under scanning electron microscopy, the fly ash particles appear as smooth, spherical particles, which is beneficial for improving the fluidity of the cementitious material paste.

## 3. Methods

### 3.1. Methods in This Study

To study the effects of fly ash content on the macro and micro properties of slag-steel slag-gypsum-based cementitious materials, the ratios of slag:steel slag:desulfurization gypsum were fixed at 52:33:15, with the addition of 0%, 5%, 10%, and 15% fly ash. The specific mix proportions are shown in Table 2. The cement-sand ratio was 1:3, the water-cement ratio was 0.3, and the superplasticizer content was 0.3% of the cementitious material’s mass. Prior to the experiment, all raw materials were ground (Ball mill purchased from Hebei Zhongke Instrument Equipment Co., Ltd. (Cangzhou, China)): slag, steel slag, and desulfurization gypsum were ground to specific surface areas of 498 m^2^/kg, 403 m^2^/kg, and 395 m^2^/kg, respectively. The raw materials, water, standard sand, and superplasticizer were mixed in a cement mortar mixer, and the fluidity of the well-mixed mortar was tested.

The mixed mortar and paste were poured into molds of sizes 40 × 40 × 160 mm and 30 × 30 × 50 mm, respectively, and kept in a standard environment at a temperature of 20 ± 2 °C and humidity of 95 ± 2% for 24 h. After demolding, the mortar and paste specimens were further cured for 3 days, 7 days, and 28 days for compressive strength and microstructure testing [31].

The compressive strength of the mortar specimens was measured according to GB/T 17671-2021 [32] and the fluidity of the mortar was measured according to GB/T 2419-2005 [33]. Microstructural test samples were soaked in alcohol to halt hydration and then dried. The samples were ground to less than 75 µm for XRD, FTIR, and TG-DSC analysis. Hardened paste specimens were broken into thin slices and cubes for SEM microstructural analysis and mercury intrusion porosimetry (MIP) pore structure analysis, respectively.

### 3.2. Characterization Methods

The chemical composition of the raw materials was analyzed using a Shimadzu XRF-1800 X-ray fluorescence spectrometer from Shimadzu Corporation of Japan (Kyoto, Japan). The mineral composition of the raw materials and the neat paste samples of the cementitious materials was analyzed using a Rigaku D/Max-RC powder X-ray diffractometer from Shimadzu Corporation of Kyoto, Japan. The X-ray source was CuKα (λ = 1.5418Å), with a testing voltage and current of 40 kV and 100 mA, respectively. The scanning angle 2θ ranged from 5° to 80°, with a scanning speed of 20°/min. The hydration process of the cementitious materials was analyzed using a Q600SDT thermogravimetric analyzer from NETZSCH of Selb, Germany, with a testing range of 20 °C to 800 °C and a heating rate of 10 °C/min [34]. The concentration of ions in the solution was determined using a DV5300 inductively coupled plasma optical emission spectrometer (ICP), following the procedure outlined in HJ 557-2009 [35]. Scanning electron microscopy analysis (SEM-EDS) was conducted using a Zeiss SUPRA 55 field emission scanning electron microscope from Oberkochen, Germany, operating at a voltage of 30 kV. The pore size distribution of the neat paste samples was determined using an Auto Pore V9620 mercury intrusion porosimeter (from the Micromeritics, Norcross, GA, USA), with a testing range of 3.2 nm to 360 µm.

## 4. Results and Discussion

### 4.1. The Impact of Fly Ash Content on the Macroscopic Properties of Cementitious Materials

**Figure 3:**
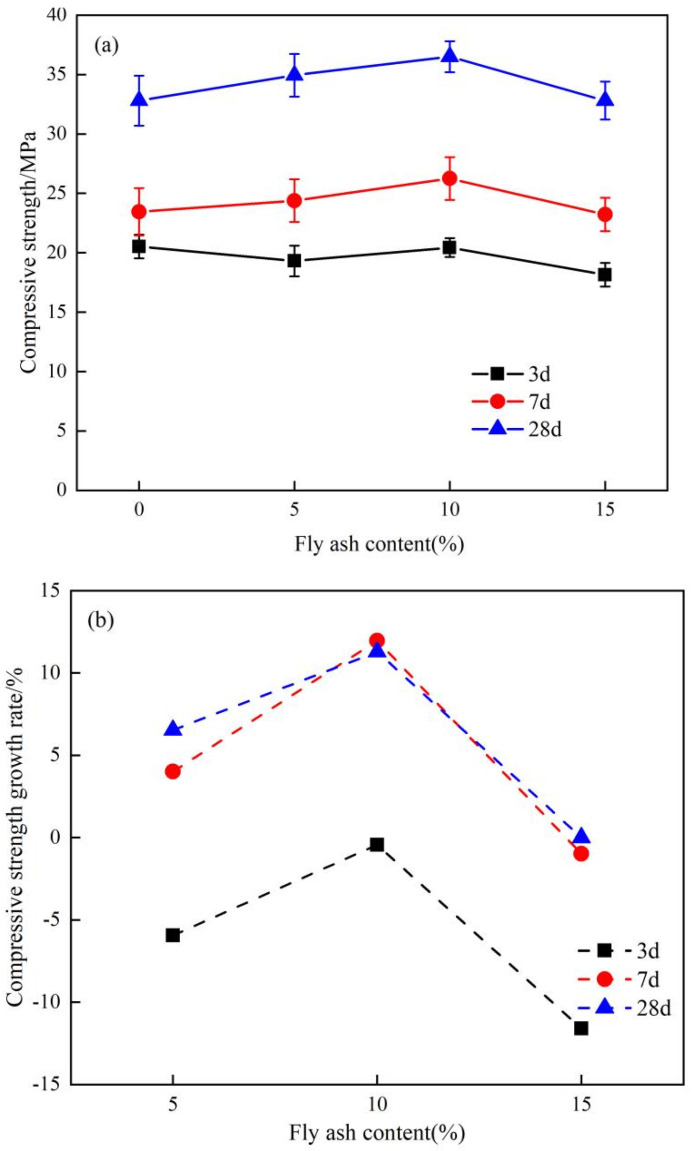
The effect of fly ash content on compressive strength. (**a**) the compressive strength, (**b**) the compressive strength rate.

Figure 3 shows the compressive strength of the cementitious materials at different ages with varying fly ash content. As shown in Figure 3a, when the fly ash content increases from 0 to 15%, the compressive strength of the cementitious materials at all ages initially increases and then decreases. When the fly ash content is 10%, the compressive strength at all ages reaches its maximum. As shown in Figure 3b, compared with the blank group, after adding 5~15% fly ash, the strength growth rate of the cementitious materials at each age also showed a trend of first increasing and then decreasing. Specifically, when the fly ash content is 10%, the compressive strength at 7 days and 28 days increases by 11.96% and 11.28%, respectively, compared to the blank group without fly ash. The main reason for the change of compressive strength of cementitious materials may be that the hydration activity of fly ash itself is weak, and the secondary hydration reaction mainly occurs in the middle and late stages. Therefore, a large amount of fly ash may lead to a decline in strength, and the dosage should be taken into account in practical applications.

Additionally, the incorporation of fly ash can improve the growth rate of compressive strength. For the control group without fly ash, the compressive strength growth rates from 3 to 7 days and from 7 to 28 days are 14.23% and 39.9%, respectively. With 10% fly ash, the corresponding growth rates are 28.46% and 41.02%. This indicates that adding fly ash to the slag-steel slag-desulfurization gypsum system has greater potential for increasing the later compressive strength of the cementitious materials. This is mainly because fly ash particles are smooth and spherical, formed at higher temperatures with a denser internal glassy structure, resulting in weaker pozzolanic activity compared to slag, and primarily occurring in the later stages of the hydration reaction. These findings are consistent with those of Han X et al. [36].

**Figure 4:**
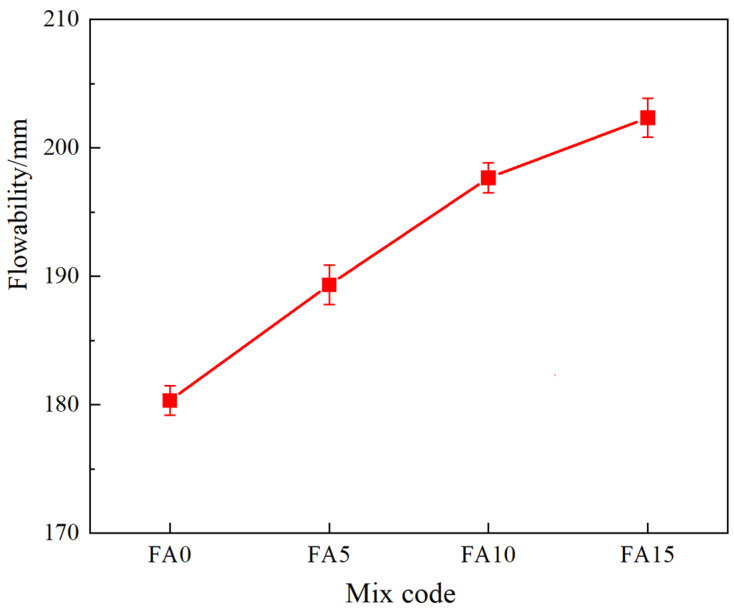
The influence of fly ash content on fluidity.

Figure 4 shows the fluidity of the cementitious materials with varying fly ash content. As the fly ash content increases, the fluidity of the cementitious materials also increases, although the rate of increase diminishes. With the addition of 15% fly ash, the fluidity is 12.8% higher than that of the control group without fly ash. This can be attributed to two main reasons: firstly, fly ash consists of smooth, dense, spherical particles with low water demand, which reduces the overall water requirement of the cementitious material. Secondly, the spherical particles of fly ash vary in size, with a D50 of 23.5 µm, allowing for continuous particle grading with slag, steel slag, and desulfurization gypsum, providing a “lubricating” effect. Consequently, the incorporation of fly ash significantly improves the fluidity of the mortar, consistent with the findings of Song [37].

**Figure 5:**
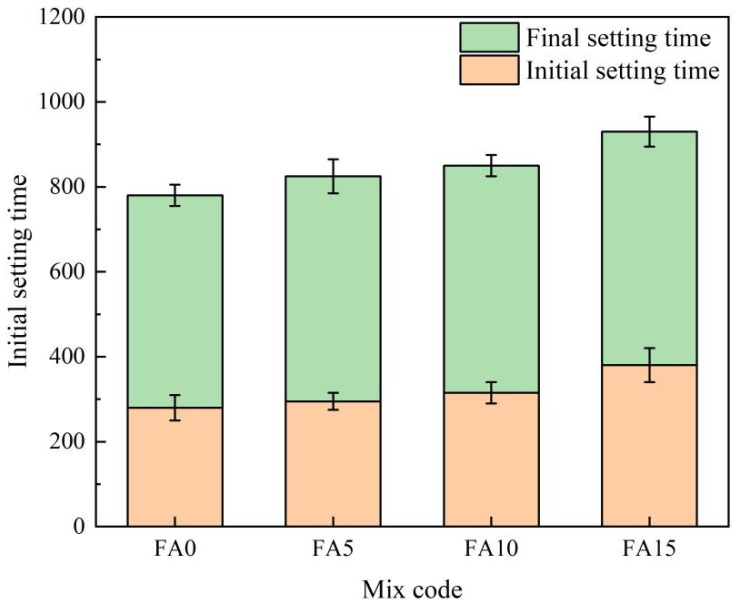
The influence of fly ash content on the setting time.

Figure 5 shows the setting time of the cementitious materials with varying fly ash content. As shown in Figure 5, with the increase of fly ash content, the initial and final setting times of the cementitious material gradually extend. When the dosage of fly ash is 10%, the initial setting time is extended by 35 min. When the dosage of fly ash is 15%, the initial setting time is extended by 100 min and the final setting time is extended by 50 min. Therefore, the more fly ash is added, the greater the impact on the setting time, and the impact on the final setting time is smaller than that on the initial setting time. The main reason for extending the setting time of fly ash is that the early activity of fly ash is low, and its spherical particles can effectively increase fluidity, but it is not conducive to the early hydration rate of cementitious materials. Since the speed of construction of the newly mixed cementitious materials depends on its setting time, it is an important task to minimize the setting time while maintaining optimal fluidity. From the results in Figure 5, it can be seen that the group FA-10 has a relatively appropriate fluidity and setting time, and thus has the best workability.

### 4.2. The Effect of Fly Ash Content on the Phase Composition of Cementitious Materials

**Figure 6:**
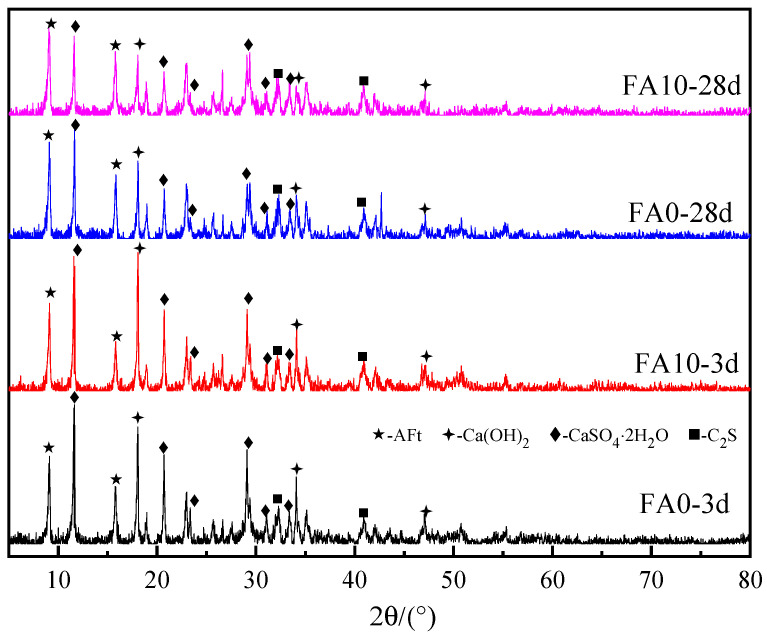
X-ray diffraction (XRD) spectra of cementitious materials based on solid waste.

Figure 6 depicts the X-ray diffraction (XRD) patterns of samples without fly ash and with 10% fly ash at different ages. It can be observed from Figure 6 that there is no significant change in the types of hydration products after the addition of fly ash. The main phases of the fly ash-slag-steel slag-gypsum solid waste-based cementitious materials are ettringite (AFt), calcium hydroxide (Ca(OH)_2_), gypsum (CaSO_4_∙2H_2_O), and dicalcium silicate (C_2_S). Before 3 days of hydration, the intensity of the ettringite diffraction peaks in samples with 10% fly ash and the control group without fly ash is almost identical. However, after 28 days of hydration, the intensity of the ettringite diffraction peaks in the samples with fly ash is significantly higher than that in the control group, while the diffraction peaks of calcium hydroxide at 18.2° and gypsum at 11.8° are lower than in the blank group. This indicates that the addition of fly ash consumes more large Ca(OH)_2_ in the liquid phase, thereby breaking the dense shell on the surface of the fly ash, resulting in the activation of pozzolanic activity [12]. After the dissolution of the silicon-oxygen tetrahedra and aluminum-oxygen tetrahedra in the fly ash, they react with Ca(OH)_2_ to form C-S-H gel and C-A-H gel. In addition, the C-A-H gel then reacts with CaSO_4_∙2H_2_O in the liquid phase to form ettringite. Therefore, the 28d diffraction peak intensity of gypsum is significantly lower than that of 3d. Both C-S-H gel and C-A-H gel are amorphous and cannot be observed in XRD patterns. Therefore, the addition of fly ash promotes the generation of hydration products in the later stages of solid waste-based cementitious materials’ hydration.

### 4.3. The Influence of Fly Ash Content on the Hydration Process of Cementitious Materials

**Figure 7:**
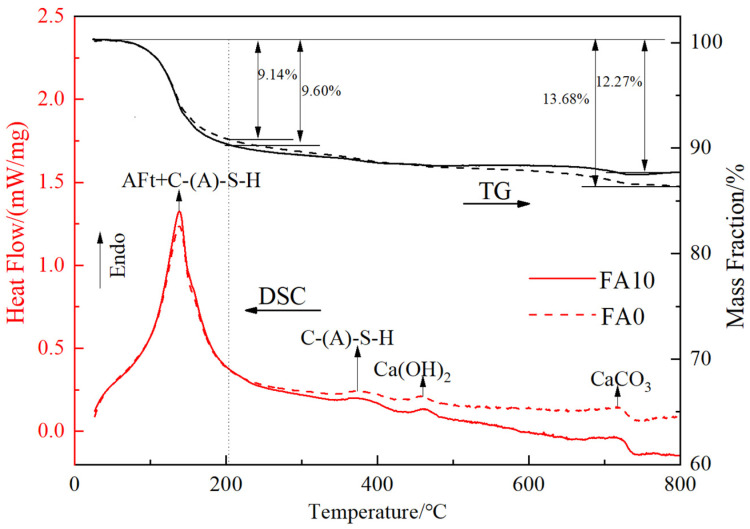
The TG-DSC curves of cementitious materials based on solid waste.

Figure 7 presents the thermogravimetric-differential scanning calorimetry (TG-DSC) curves of samples without fly ash and with 10% fly ash at 28 days. In the TG-DSC curves shown in Figure 7, the positions of the significant endothermic peaks for both types of solid waste-based cementitious materials are basically the same after the addition of fly ash, indicating that their mineral compositions are similar, which is consistent with the XRD results. The significant endothermic peak at 138 °C is attributed to the dehydration of ettringite (AFt) and the loss of water from C-S-H gel/C-A-H gel [38]. Before 200 °C, the mass loss rates of samples with 10% fly ash and the control group without fly ash are 9.14% and 9.60%, respectively. Combined with the diffraction peak corresponding to AFt at 8.3° in the XRD pattern, this suggests that the addition of fly ash increases the formation of hydration products such as ettringite (AFt) and C-S-H gel/C-A-H gel at 28 days. The weak endothermic peak between 350–400 °C is caused by the decomposition of C-S-H gel and C-A-H gel. The endothermic peak between 400–500 °C is attributed to the decomposition of Ca(OH)_2_ [39], with the peak height of FA10 significantly lower than that of FA0, indicating that the addition of fly ash consumes more Ca(OH)_2_ in the liquid phase and promotes the activation of fly ash’s pozzolanic activity, thereby facilitating the formation of C-S-H gel and C-A-H gel. The endothermic peak near 700 °C is caused by the dehydroxylation of CaCO₃ [40]. The mass loss rates of FA10 and FA0 between 25–800 °C are 12.27% and 13.68%, respectively, mainly due to the higher content of Ca(OH)_2_ and CaCO_3_ in the solid waste-based cementitious materials without fly ash. Therefore, the addition of fly ash facilitates the generation of hydration products and the consumption of Ca(OH)_2_ and CaCO_3_ in the raw materials of the solid waste-based cementitious materials.

### 4.4. The Influence of Fly Ash Content on the Leaching Ion Concentration of Cementitious Materials

**Figure 8:**
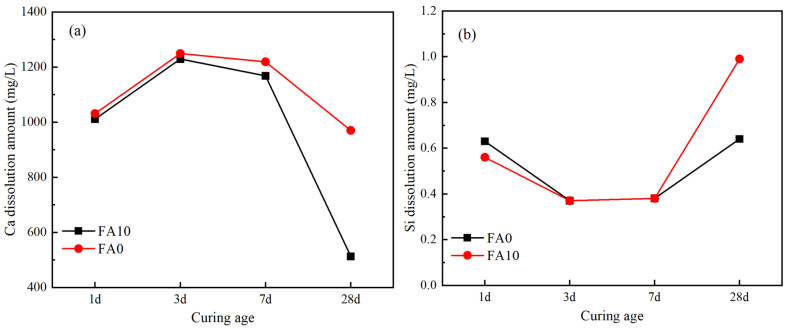
The leaching ion concentration of solid waste-based cementitious materials. (**a**) Ca dissolution amount, (**b**) Si dissolution amount.

Figure 8 illustrates the ion-leaching concentration of samples without fly ash and with 10% fly ash at different ages. According to the test method in HJ-557 [35], samples of FA0 and FA10 at various ages were pre-treated before leaching, and then the concentrations of Ca and Si leachates in the net slurry samples at each age were measured using ICP-MS to observe the influence of fly ash addition on the leaching of various elements in the cementitious materials.

As shown in Figure 8a, with the extension of curing time, the concentration of Ca leachate undergoes a process of initial increase followed by decrease. The changing trend of FA0 and FA10 at 7 days of hydration is similar, indicating that the influence of fly ash addition on the early hydration is small, which is consistent with the XRD results. In the early hydration stage, a large amount of Ca leaches out into the liquid phase, mainly existing in the form of Ca^2+^ in the pore solution, and some Ca^2+^ participates in the hydration reaction of slag, generating ettringite (AFt), C-S-H, and C-A-H gels. However, since the overall rate of Ca^2+^ leaching is greater than its consumption rate, there is a significant increase in the concentration of Ca^2+^ in the liquid phase. However, in the later stage of hydration, with the continuous decrease in the Ca(OH)_2_ content in the system and the continuous generation of various hydration products, the consumption rate of Ca^2+^ in the liquid phase exceeds the leaching rate, resulting in a continuous decrease in the concentration of Ca^2+^ in the slurry. Especially after adding fly ash, the decrease in the concentration of Ca^2+^ in the later stage of hydration is more significant, indicating that fly ash mainly promotes the later hydration reaction in the slag-steel slag-gypsum cementitious materials [41].

As shown in Figure 8b, the concentration of Si leachate undergoes a process of initial decrease followed by increase. Si mainly migrates in the liquid phase in the form of silicate tetrahedra or silicate tetrahedral groups. In the early hydration stage, the Si leached out from the slag quickly reacts with the abundant Ca^2+^ and OH^−^ in the liquid phase to form C-S-H gel, leading to a rapid decrease in the concentration of Si leachate. However, in the later stage of hydration, while the concentration of Ca^2+^ in the liquid phase decreases, the concentration of Si leachate increases significantly. Especially after adding fly ash, the increase in the concentration of Si leachate is more pronounced. This is mainly because the pozzolanic activity of fly ash is low, and it can disintegrate and leach out silicate tetrahedra only in the later stage of hydration [42].

Therefore, the addition of fly ash has little effect on the leachate concentration of Ca and Si in the early stages of the cementitious system, but after 7 days, its dissolution rate begins to accelerate, continuously dissolving out silicate tetrahedra or aluminate tetrahedral groups, while simultaneously consuming Ca^2+^ and OH^−^ in the system and generating C-S-H gel and ettringite (AFt). Therefore, the experimental group with fly ash addition can generate more hydration products, which is consistent with the analysis results of XRD and TG-DSC.

### 4.5. The Influence of Fly Ash Content on the Microstructure of Cementitious Materials

**Figure 9:**
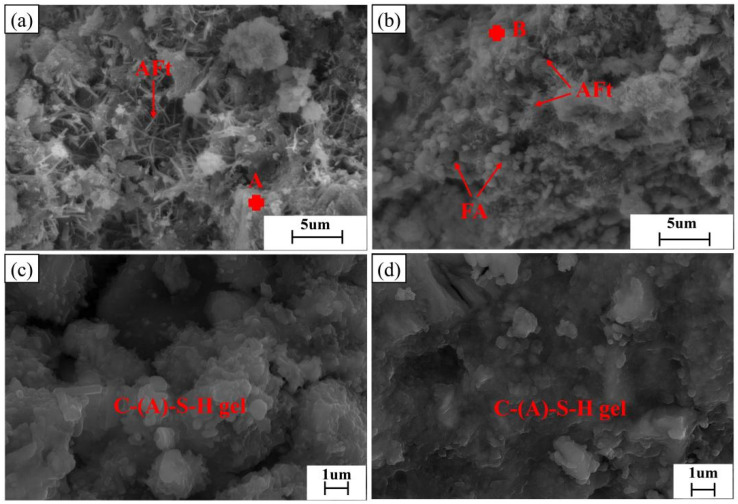
The 28d SEM photos of solid waste-based cementitious materials: (**a**) FA0-28d; (**b**) FA10-28d; (**c**) Amplification area of point A; (**d**) Amplification area of point B.

Figure 9 shows the scanning electron microscope (SEM) images of samples without fly ash and with 10% fly ash after 28 days of hydration. According to the energy-dispersive X-ray spectroscopy (EDS) analysis, the needle-like crystals are ettringite (AFt).

As seen in Figure 9a,b, a large amount of ettringite (AFt) is formed after 28 days of hydration in both samples, with and without fly ash. However, the difference lies in the size and morphology of the ettringite (AFt) [27]. In the sample without fly ash, the formed ettringite (AFt) is coarser, while in the sample with 10% fly ash, the ettringite (AFt) is enveloped by flocculent phases, and the exposed ettringite is finer. Meanwhile, there are many obvious pores and voids on the surface of the sample without fly ash, whereas in FA10, spherical particles of fly ash can be seen filling the voids, making the overall structure denser. This indicates that the addition of fly ash to the slag-steel slag-gypsum system can not only increase the hydration products in the later stage but also play a role in physical filling, reducing the generation of cracks and weak areas.

Upon magnifying regions A and B in Figure 9a,b, it can be observed that the flocculent phases mainly consist of C-(A)-S-H gel. The difference lies in the C-(A)-S-H gel formed after adding fly ash, which exhibits high-polymerized granules interconnected into a whole structure, whereas the gel without fly ash shows a loosely aggregated granular structure with low polymerization [43].

**Figure 10:**
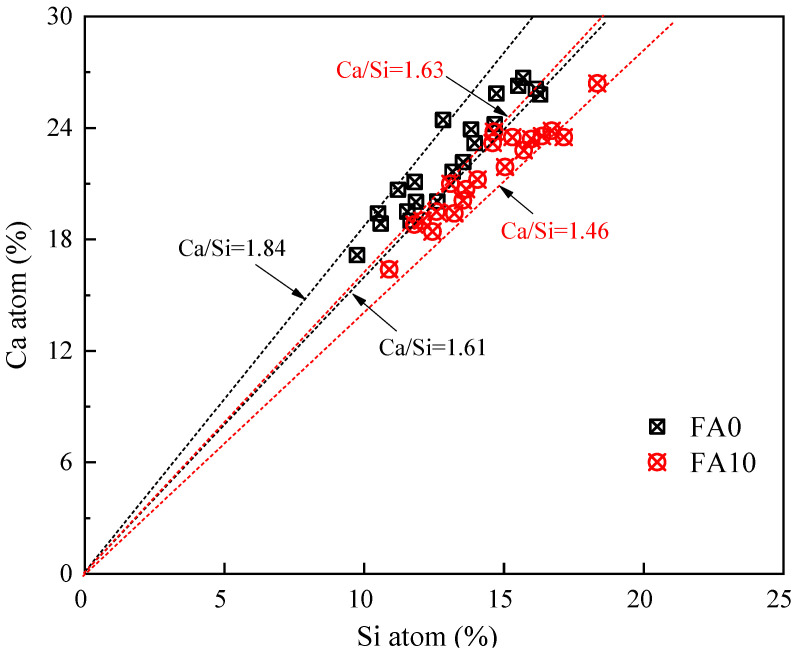
Ca/Si of solid waste-based cementitious materials C-(A)-S-H gel.

To study the effect of fly ash on the hydration product C-(A)-S-H gel in the solid waste-based cementitious materials, EDS analysis was conducted on 20 points in the gel regions of Figure 9c,d. The atomic ratio of Ca to Si in the C-(A)-S-H gel was obtained, as shown in Figure 10.

For the sample without fly ash, the range of Ca/Si (atomic ratio) in the C-(A)-S-H gel was 1.61 to 1.84. On the other hand, for the sample with 10% fly ash, the range of Ca/Si (atomic ratio) in the C-(A)-S-H gel was 1.46 to 1.63. This indicates that the addition of fly ash can reduce the Ca/Si ratio of the C-(A)-S-H gel [26]. C-(A)-S-H gel with a lower Ca/Si ratio exhibits longer silicate chains, and gels with lower Ca/Si ratios have higher mechanical properties [26,43]. Therefore, fly ash improves and enhances the structure and microstructure of hydration products in slag-steel slag-gypsum cementitious materials, thereby exhibiting higher mechanical properties at the macro level.

## 5. Discussion

Fly ash has a significant impact on the macroscopic workability, mechanical properties, microscopic hydration products, hydration process, and microstructure of solid waste-based cementitious materials. Therefore, based on relevant cutting-edge research, this chapter conducts further discussion and analysis.

1. The impact on macroscopic performance

The macroscopic properties of cementitious materials mainly include fluidity, setting time, and compressive strength. Firstly, in terms of flowability, the addition of 5% to 15% fly ash increases the flowability of cementitious materials. Research has shown that fly ash is a smooth and dense spherical particle as a whole, and its addition can reduce the water demand of cementitious materials [37]. At the same time, the continuous gradation of various mineral admixtures can improve the flowability of cementitious materials, especially the D50 of fly ash in this article is 23.5 µm, which is more conducive to increasing the flowability of the materials [15]. Secondly, in terms of setting time performance, the addition of 5% to 15% fly ash prolongs the initial and final setting times of solid waste-based cementitious materials. The activity index of II and fly ash is generally 50~75%, which is significantly lower than that of S95 and S105 slag powder [44]. Therefore, the higher the substitution amount of fly ash, the slower the hydration rate of the cementitious material and the longer the setting time.

In addition, the low activity of fly ash can also affect the compressive strength performance of cementitious materials. After adding 5% to 15% fly ash, the compressive strength of solid waste-based cementitious materials shows a trend of first increasing and then decreasing, with the optimal dosage being 10%. However, the addition of fly ash has a significant impact on improving the compressive strength in the middle and later stages. This is mainly because the glass-like structure inside fly ash is relatively dense, and the secondary hydration reaction with calcium hydroxide mainly occurs in the middle and later stages [36]. In addition, the spherical particles of fly ash fill the pores in the paste, resulting in a denser overall structure and reducing the formation of cracks and weak areas.

2. The impact on microscopic products and structures

Fly ash appears as spherical particles of varying sizes under scanning electron microscopy, mainly composed of Al_2_O_3_, SiO_2_, CaO, Fe_2_O_3_, TiO_2_, MgO, Na_2_O, and other components. There are a large number of hydroxyl groups on the surface of fly ash particles, which have good permeability in a loose state and therefore have potential volcanic ash activity [16].

Research has shown that the high alkalinity of steel slag can effectively stimulate the volcanic ash activity of fly ash and promote the breaking of active Si-O-Si, Si-O-Al, and Al-O-Al bonds inside fly ash. Depolymerization occurs to form [H_3_SiO_4_]^−^ tetrahedra, [H_3_AlO_4_]^2−^ tetrahedra, and [Al(OH)_6_]^3−^ octahedra [42]. In alkaline solution, when saturated concentrations of Ca^2+^, SO_4_^2−^, active SiO_2_, and Al_2_O_3_ ions are present in the alkaline liquid phase, the [Al(OH)_6_]^3−^octahedron rapidly crystallizes with Ca^2+^, SO_4_^2−^ to form an extremely low solubility complex salt—AFt($3\mathrm{CaO}\cdot {\mathrm{Al}}_{2}O_{3}\cdot 3\mathrm{CaS}O_{4}\cdot 32H_{2}O$). At the same time, the [H_3_SiO_4_]^−^ tetrahedron and [H_3_AlO_4_]^2−^ tetrahedron in the liquid phase react with Ca^2+^ to generate C-S-H gel and C-A-H gel [45,46]. In addition, the addition of fly ash significantly reduced the Ca/Si ratio of C-(A)-S-H gel, thus increasing the degree of polymerization of hydrated C-(A)-S-H gel, which is conducive to improving the macro mechanical strength.

## 6. Conclusions

This study investigates the development of a novel cement-free quaternary solid waste-based cementitious materials using four types of solid waste: fly ash (FA), granulated blast furnace slag (GGBS), steel slag (SS), and desulfurization gypsum (DG). The research focuses on examining the influence of fly ash on the macro-performance and microstructure of the quaternary cementitious material. The optimal FA content in the cementitious material is 10%, resulting in a 28-day compressive strength of the mortar specimens that is 11.28% higher than that of the control group. The inclusion of FA reduces the overall water demand of the cementitious material, thereby enhancing the workability of fresh mortar, with this improvement being positively correlated with the FA content.

The primary hydration products of the FA-GGBS-SS-DG-based cementitious material are ettringite (AFt) and C-(A)-S-H gel. FA has minimal impact on the early hydration products and processes of the cementitious material but contributes to the increased formation of AFt and C-(A)-S-H gel in the hardened paste during later stages of hydration. In the quaternary solid waste-based cementitious materials, SS plays a crucial role by providing alkalinity during the hydration reaction of FA. Ion-leaching results confirm that FA has a limited effect on the early-release concentrations of calcium and silicon in the cementitious system, but their concentrations increase after 7 days. The addition of FA lowers the Ca/Si ratio of the C-(A)-S-H gel, thereby enhancing the degree of polymerization of the hydrated C-(A)-S-H gel, which contributes to improved macroscopic mechanical strength. Furthermore, the spherical particles of FA fill the pores in the paste, resulting in a denser overall structure that reduces the formation of cracks and weak areas.

The findings of this study offer valuable insights for the synergistic utilization of multiple solid wastes in producing low-carbon, cement-free solid waste-based cementitious materials, with significant economic and environmental benefits. However, due to the limitations of the research scope and article length, this paper primarily focuses on analyzing the effective role of FA in the solid waste-based cementitious materials. Future research should further explore the effects of the various components on material performance and the hydration-driven mechanisms within the quaternary solid waste-based cementitious materials.

## Figures and Tables

**Table 1:** Chemical composition of the raw materials (mass/%).

	CaO	SiO_2_	Al_2_O_3_	Fe_2_O_3_	MgO	P_2_O_5_	MnO	TiO_2_	SO_3_
Slag	39.37	29.83	14.22	0.36	8.97	0.02	0.17	3.91	2.45
Steel slag	53.46	9.85	3.43	17.97	6.49	0.88	1.17	-	3.96
Gypsum	42.64	1.87	0.52	0.51	0.79	-	0.02	-	35.57
Fly ash	5.74	38.6	28.0	4.06	0.73	-	0.07	1.14	0.59

**Table 2:** The mixture ratio of fly ash-slag-steel slag-gypsum solid waste-based cementitious materials.

	Materials	Solid Waste-Based Cementitious Materials				Water/g	ISO Sand/g	Admixture/g
Number		Slag	Steel Slag	Gypsum	Fly Ash			
FA0		52%	33%	15%	0	135	1350	1.35
FA5		52%	33%	15%	5%	135	1350	1.35
FA10		52%	33%	15%	10%	135	1350	1.35
FA15		52%	33%	15%	15%	135	1350	1.35

## Data Availability

The original contributions presented in the study are included in the article, further inquiries can be directed to the corresponding authors.
